# 24.1 NEUROCOGNITIVE DEVELOPMENT FROM INFANCY TO EARLY ADULTHOOD IN THE PSYCHOSIS SPECTRUM

**DOI:** 10.1093/schbul/sby014.096

**Published:** 2018-04-01

**Authors:** Josephine Mollon, Anthony David, Stanley Zammit, Glyn Lewis, Abraham Reichenberg

**Affiliations:** 1 Yale University; 2 Institute of Psychiatry, King’s College London; 3 Cardiff University; 4 University College London; 5 Icahn School of Medicine at Mount Sinai

## Abstract

**Background:**

The majority of patients with psychotic disorders experience severe neuropsychological impairment. The onset and course of this impairment, however, is debated. Moreover, the course of neuropsychological functioning in other psychiatric conditions remains largely unexamined. This study used longitudinal data from infancy to early adulthood to chart the course of general and specific neuropsychological functions in individuals with psychotic disorders, psychotic experiences and depression.

**Methods:**

Data were from the Avon Longitudinal Study of Parents and Children (ALSPAC), a prospective cohort study comprising all live births between 1991 and 1992 in Avon, UK. All participants who underwent cognitive testing at 18 months, 4, 8, 15 and 20 years, and psychiatric assessment at age 18 were included. Individuals with non-affective psychotic disorder, affective psychotic disorder, subclinical psychotic experiences and depression were compared to controls on full-scale, verbal and non-verbal IQ, and measures of processing speed, working memory, language, visuospatial ability and attention.

**Results:**

Individuals with non-affective psychosis showed continually increasing deficits between infancy (18 months) and adulthood (20 years) in full-scale IQ (effect size of change (ESΔ) =−1.09, p=.02), and non-verbal IQ (ESΔ=−0.94, p=.008). The depression group showed a small, increasing deficit in non-verbal IQ (ESΔ=−0.29, p=.04) between infancy and adulthood. Between ages 8 and 20, the non-affective psychosis group exhibited developmental lags (i.e. slower growth) on measures of processing speed, working memory and attention (ESΔ=−0.68, p=.001; ESΔ=−0.59, p=.004; ESΔ=−0.44, p=.001), and large, static deficits on measures of language and visuospatial ability (ES=−0.87, p=.005; ES=−0.90, p=.001). There was only weak evidence for neuropsychological deficits in individuals with affective psychosis, depression, and subclinical psychotic experiences.

**Discussion:**

These findings suggest that the origins of non-affective psychotic disorder involve dynamic neurodevelopmental processes, which effect both verbal and non-verbal abilities throughout the first two decades of life. These neurodevelopmental processes do not manifest in other psychiatric disorders, such as affective psychotic disorder and depression.

